# Involvement of EGFR-AKT signaling in hemin-induced neurotoxicity

**DOI:** 10.3389/ebm.2025.10554

**Published:** 2025-05-15

**Authors:** Hui-Ju Huang, Yang-Jie Tseng, I-Jung Lee, Yu-Li Lo, Anya Maan-Yuh Lin

**Affiliations:** ^1^ Department of Medical Research, Taipei Veterans General Hospital, Taipei, Taiwan; ^2^ Ph.D. Program in Regulatory Science and Policy, National Yang-Ming Chiao-Tung University, Taipei, Taiwan; ^3^ Pharmaceutical Botany Research Laboratory, Yokohama University of Pharmacy, Yokohama, Japan; ^4^ Institute of Pharmacology, National Yang-Ming Chiao-Tung University, Taipei, Taiwan; ^5^ Department of Pharmacy, National Yang-Ming Chiao-Tung University, Taipei, Taiwan

**Keywords:** hemin, EGFR-AKT signaling, afatinib, primary cultured cortical neurons, ferroptosis

## Abstract

Intracerebral hemorrhage (ICH), as bleeding from ruptured vessels within the brain, is the second leading neuropathological problem following ischemic stroke. In the present study, the involvement of epithelial growth factor receptor (EGFR)-tyrosine kinase (TK) signaling underlying ICH-related neurodegeneration was investigated using afatinib, a clinically available EGFR-tyrosine kinase inhibitor (EGFR-TKI). We employed hemin (a breakdown product of hemoglobin) to mimic the pathophysiology of ICH in primary cultured cortical neurons. Using a lactate dehydrogenase (LDH) assay, incubation of hemin concentration- and time-dependently induced neuronal death. Simultaneous incubation of afatinib (10 nM) significantly inhibited hemin (30 μM)-induced neuronal death. Immunofluorescent data demonstrated that co-treatment of afatinib for 1 h attenuated hemin (30 μM)-induced elevation in phosphorylated-EGFR (p-EGFR) immunoreactivity and neurite impairment. Western blot assay demonstrated that co-incubation of afatinib for 16 h diminished hemin-induced elevation in p-EGFR and p-AKT, tumor necrosis factor-α and cyclooxygenase 2 (two proinflammatory biomarkers) as well as heme oxygenase-1 (HO-1, an enzyme catalyzing heme/hemin), glutathione hydroperoxidase 4 and receptor-interacting protein 3 (two biomarkers of ferroptosis and necroptosis). In addition, co-treatment of afatinib for 24 h inhibited hemin-induced NO production in the culture medium. In conclusion, our study shows that afatinib via blocking EGFR-AKT signaling inhibits hemin-induced EGFR-AKT activation, neuroinflammation, HO-1 expression and programed cell death, suggesting that EGFR-AKT signaling is involved in hemin-induced neurotoxicity and may be a druggable target for ICH.

## Impact statement

To find potential therapies for the secondary injury in ICH, the involvement of EGFR-TK signaling in ICH was investigated *in vitro*. We found that hemin induced neuronal death as well as EGFR-AKT activation, neuroinflammation, HO-1 expression, ferroptosis and necroptosis in primary cultured cortical neurons. Furthermore, afatinib, a clinically available EGFR-TK inhibitor, is capable of blocking hemin-induced EGFR-AKT activation and neurotoxicity. Our data suggest that EGFR-AKT signaling may be a druggable target for ICH.

## Introduction

Intracerebral hemorrhage (ICH) is due to the rupture of blood vessels in the parenchyma. ICH reportedly induces primary and secondary brain injuries [1, 2]. The primary injury is initiated acutely by local pressure and mechanical damage due to hematoma in the brain. The secondary injury results from released blood which is degraded to blood derived products, including heme/hemin and iron. These blood-derived products reportedly induce oxidative injury, neuroinflammation and protein aggregation that lead to cell death [1, 3–5]. Current therapies for the primary injury include surgical removal of blood clots and drug treatment to reduce the elevated intracranial pressure [5]. Neuroprotective strategies toward the secondary injury are urged.

Cellular growth factors and its kinase pathways, such as epidermal growth factor receptor (EGFR) [6], have been used as cancer therapies for decades [7, 8] and are now proposed for central nervous system (CNS) neurodegenerative diseases [9, 10]. Physiologically, EGFR is involved in the developing nervous system by regulating growth, differentiation and repair [9]. In a healthy adult brain, EGFR is expressed in specific regions, such as the subventricular zone [11]. However, a clinical report showed intensive EGFR expression in the neurites in affected brain tissues of patients with Alzheimer disease (AD), suggesting an EGFR reactivation in response to insults [12]. In support of this notion, an Alzheimer’s pre-clinical study demonstrated that EGFR inhibitors ameliorated Aβ42-induced neurotoxicity [13]. Furthermore, both AG1478 (an EGFR antagonist) and C225 (anti-EGFR monoclonal antibody) reportedly reduced the expression of phosphorylated EGFR (p-EGFR), enhanced axonal outgrowth and promoted functional recovery in rats subjected to spinal cord injury (SCI) [14]. Moreover, AZD 3759, a blood brain barrier permeable EGFR-tyrosine kinase inhibitor (EGFR-TKI), was found to reduce the phosphorylated α-synuclein levels, a pathological biomarker of Parkinson’s Disease [10]. Our previous study showed that afatinib, a clinically available EGFR-TKI for lung cancer therapy, attenuated oxygen-glucose deprivation (OGD)-induced neuroinflammation in primary cultured astrocytes [15]. Accordingly, a pathological role of EGFR signaling is suggested in CNS neurodegenerative diseases.

Many studies have focused on the involvement of EGFR in ischemic stroke [16–18]. However, only one pre-clinical study demonstrated that AG1478 inhibited neuronal apoptosis in mice subjected to subarachnoid hemorrhage [19]. In the present study, the involvement of EGFR in the pathophysiology of ICH was investigated using hemin to mimic ICH-related neurotoxicity [20, 21]. Moreover, afatinib, via binding to the EGFR-ATP binding activation site [22], was repurposed to block hemin-induced EGFR activation and hemin-induced neurotoxicity in neurons to delineate the EGFR-AKT signaling in the pathophysiology of ICH.

## Materials and methods

### Chemicals

The chemicals used were hemin (Sigma, St. Louis, MO, United States) and afatinib (AdooQ Bioscience, Irvine, CA, United States). Hemin was dissolved in ammonia water and pH value was corrected to pH 7.4. Afatinib was dissolved in dimethyl sulfoxide (DMSO, Sigma) and diluted with DMEM or Neurobasal (NB, Thermo Fisher Scientific, Waltham, MA, United States) medium.

### Animals

Twenty-three pregnant female Sprague-Dawley (SD) rats were supplied by BioLASCO Taiwan Co., Ltd. (Yilan, Taiwan). All animals (one rat/individually ventilated cage) were housed in an air-conditioned room (22 ± 2°C) on a 12 hr-light/dark cycle (07:00–19:00h light) and had free access to food and water. Pregnant female Sprague-Dawley (SD) rats of 17-day gestation were sacrificed by an overdose of Zoletil^®^ (Virbac, Taiwan) to minimize pain or discomfort. Embryonic day 17 fetal rat brains obtained from pregnant female SD rats were used to prepare primary cultured cortical neurons. The use of animals and all experiments conducted were under approved protocols from the Institutional Animal Care and Use Committee (IACUC) of Taipei Veterans General Hospital, Taipei, Taiwan. The approval number is IACUC2022-235. All experiments were performed in accordance with relevant guidelines and regulations.

### Primary culture of cortical neurons

Cerebral cortices of fetal rats were isolated and dissociated mechanically. The dissociated cells were suspended in the Basal Medium Eagle (BME, Thermo Fisher Scientific) medium containing 20% fetal bovine serum, and were seeded onto 35-mm culture dishes (IWAKI, Tokyo, Japan) with a density of 5 × 10^6^ cells per dish. Afterwards, cells were maintained with serum-free Neurobasal medium supplemented with B27 (Thermo Fisher Scientific) in the incubator with 5% CO_2_ at 37°C. Four experimental treatments included vehicle (as control), hemin (30 μM), hemin (30 μM) plus afatinib (10 nM) and afatinib (10 nM).

### Cytotoxicity assay

In brief, primary cultured cortical neurons were seeded on a 24-well plate. Concentration-dependent effects (10–60 μM) of hemin were performed for 16 and 24 h. The effect of afatinib was investigated 16 h after simultaneous addition of afatinib (10 nM) and hemin (30 μM). Cytotoxicity was determined by a Lactate Dehydrogenase (LDH) assay. The LDH released in the culture medium was assessed by adding β-nicotinamide adenine dinucleotide and sodium pyruvate (Sigma). LDH activity was determined by measuring the absorbance at 340 nm for 6 min using an enzyme-linked immunosorbent assay (ELISA) reader (TECAN Sunrise, Männedorf, Switzerland). The LDH activity of cells treated with 0.1% Triton X-100 was used as control set to 100%.

### Western blots analysis

At the end of 16-h treatments, the cells were collected, washed with phosphate buffered saline (PBS), and lysed in a radioimmunoprecipitation assay (RIPA, Cell Signaling Tech., Beverly, MA, United States) lysis buffer containing 20 mM Tris HCl, 150 mM NaCl, 1% (v/v) NP-40, 1% (w/v) sodium deoxycholate, 1 mM ethylenediaminetetraacetates (EDTA), 0.1% (w/v) sodium dodecyl sulfate polyacrylamide (SDS) and 0.01% (w/v) sodium azide (pH 7.5) for 20 min on ice. Lysates were then centrifuged at 13,800x*g* for 10 min, and the protein concentrations of supernatants were determined by Pierce BCA Protein Assay Kit (Thermo Fisher Scientific). Protein samples (30 μg) were run on 12%–13.5% SDS-polyacrylamide gel electrophoresis and then transferred onto a polyvinylidene difluoride (PVDF, Bio-Rad, Hercules, CA, United States) at 100 V for 120 min. Blots were probed with primary antibodies including antibodies against p-EGFR/total-EGFR, p-AKT/total-AKT (Cell Signaling Tech.), Tumor necrosis factor (TNF)-α, Cyclooxygenase 2 (COX2), Heme oxygenase-1 (HO-1) (StressGen, Victoria, CA, United States), Glutathione hydroperoxidase 4 (GPX4) and Receptor-interacting serine/threonine-protein kinase 3 (RIP3) (Cell Signaling Tech.) overnight at 4°C. After incubation of primary antibodies, the membrane was washed and incubated with a secondary antibody for 1 h at room temperature. The secondary antibodies were horseradish peroxidase-conjugated secondary IgG (Chemicon, Temecula, CA, United States). The immunoreaction was visualized using Amersham Enhanced Chemiluminescence (Amersham Pharmacia Biotech, Piscataway, NJ, United States). After this measurement, the bound primary and secondary antibodies were stripped by incubating the membrane in stripping buffer (100 mM 2-mercaptoethanol, 2% SDS) at 50°C for 5 min. The membrane was reprobed with a primary antibody against β-actin (Millipore, Billerica, MA, United States).

### Immunofluorescent staining

At the end of 1-hr treatments, the cells were fixed with 4% paraformaldehyde (Merck, Boston, MA, United States). Cells were then washed with 0.1 M PBS, incubated with 0.3% Triton X-100 (Sigma) and 1% goat serum (GS; Jackson ImmunoResearch, West Grove, PA, United States), and blocked with 3% GS for 60 min. Next, cells were processed for immunostaining using mouse monoclonal antibody specific for rat p-EGFR and microtubule-associated protein 2 (MAP-2, Millipore) in 1% GS-PBS at 4°C for 24 h. The cells were then incubated in fluorescein conjugated-IgG (FITC) (Jackson ImmunoResearch) and Texas Red dye-conjugated IgG fraction monoclonal mouse anti-biotin (Jackson ImmunoResearch) for 1 h at room temperature, mounted in glycerol (Merck). Controls consisted of omission of primary antibodies. The sections were visualized by a fluorescence confocal microscope (Olympus FluoView, Norfolk, VA, United States).

### Nitric oxide (NO) production

At the end of 24-h treatments, the culture medium was collected to measure NO production. The culture medium was mixed with an equal volume of the Griess reagent (1% sulfanilamide, 0.1% N-(1-Naphthyl)ethylenediamine in 2.5% H_3_PO_4_) and incubated for 15 min at room temperature in the dark. Nitrite concentration was determined by measuring the absorbance at 550 nm using an ELISA plate reader (TECAN Sunrise, Männedorf, Schweiz).

### Statistics

All data are expressed as the mean ± standard error of the mean (S.E.M.). The results were analyzed by one-way analysis of variance (one-way ANOVA) followed by the least significance difference (LSD) test as post-hoc method. The significance level was set at p < 0.05.

## Results

### Afatinib attenuated hemin-induced neuronal death

**FIGURE 1:**
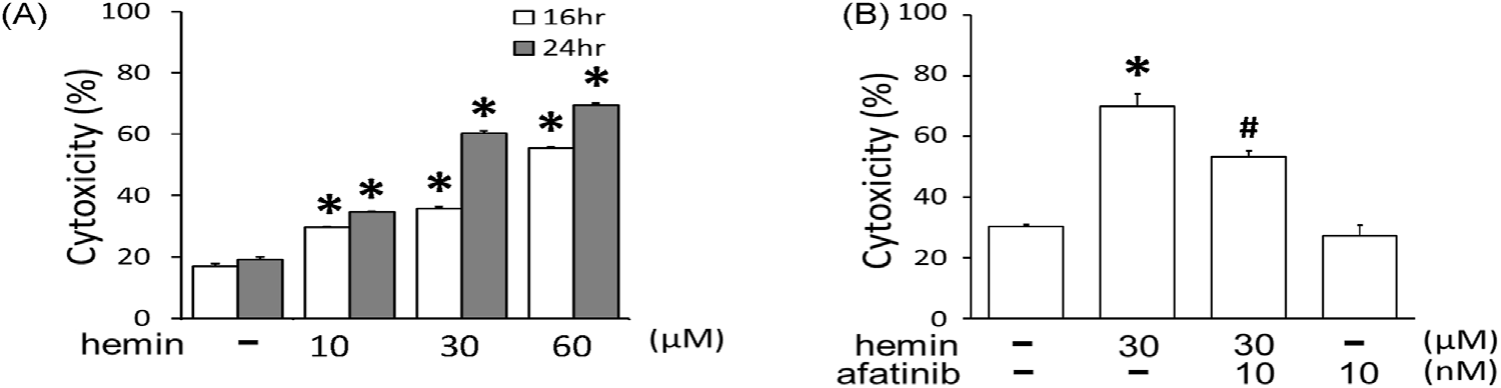
Effects of afatinib on hemin-induced cytotoxicity. **(A)** Primary cultured cortical neurons were treated with hemin (10–60 μM) for 16 h and 24 h. **(B)** Primary cultured cortical neurons were treated with hemin (30 μM) plus afatinib (10 nM) simultaneously for 16 h. Cell death was measured by LDH assay. Values are the mean ± S.E.M. (n = 3/each group). *p < 0.05 statistically significant in the hemin groups compared with the control groups; ^#^P < 0.05 in hemin plus afatinib compared with hemin alone by one-way ANOVA followed by the LSD test as post-hoc method.

To mimic the neurodegeneration in ICH, a hemin-induced neurotoxicity model was established in primary cultured cortical neurons. The LDH assay showed that incubation of hemin (10–60 μM) for 16 and 24 h increased neuronal death in time- and concentration-dependent manners (Figure 1A). The IC_50_ of hemin in primary cortical neurons was about 30 μM after 24-hr incubation. Co-incubation with afatinib (10 nM) for 16 h significantly attenuated hemin-induced cell death in primary cultured cortical neurons (Figure 1B), indicating that afatinib is capable of inhibiting hemin-induced neuronal death.

### Afatinib attenuated hemin-induced EGFR-AKT activation and morphological changes

**FIGURE 2:**
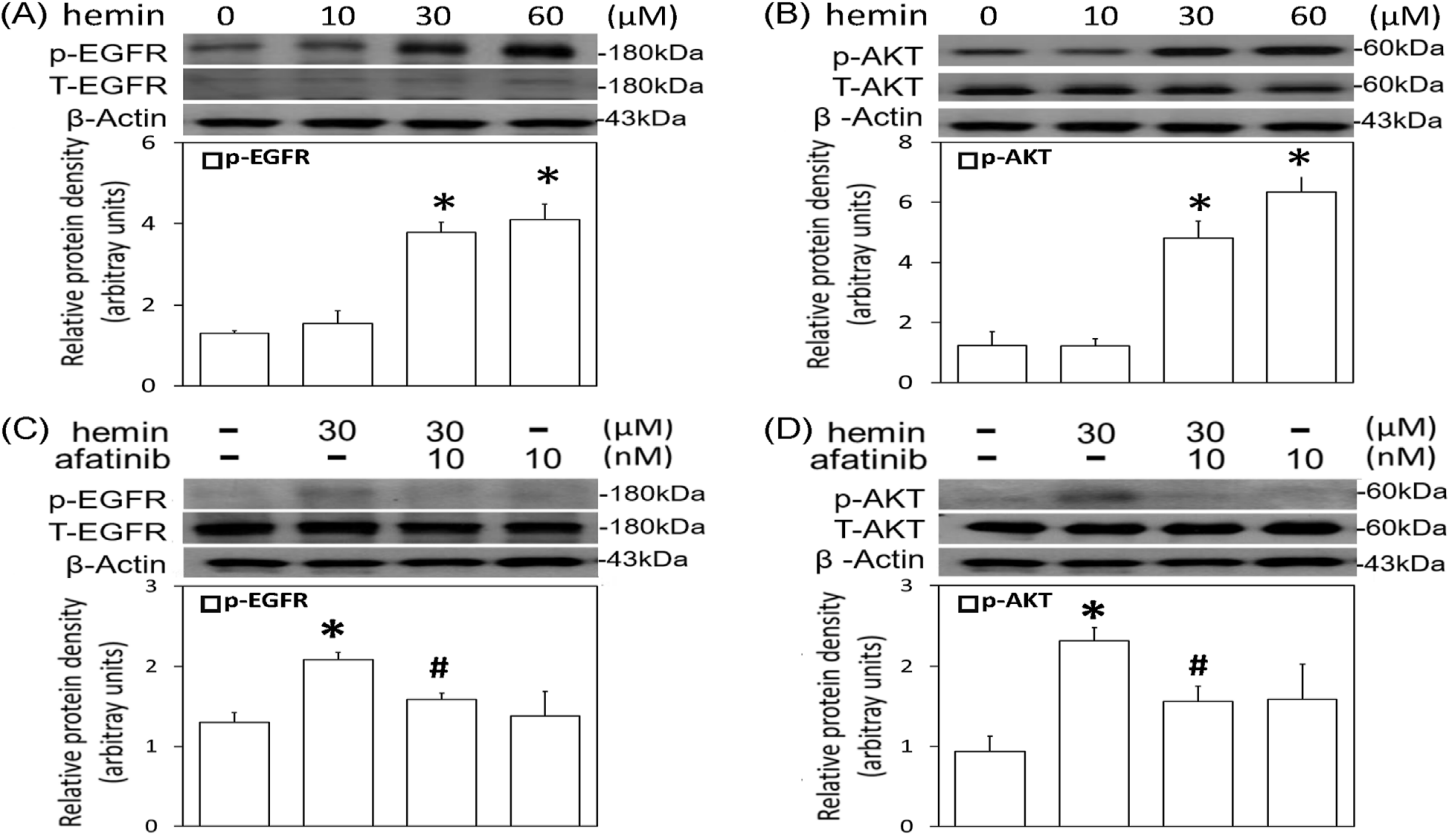
Effects of afatinib on hemin-induced EGFR-AKT activation. **(A,B)** Primary cultured cortical neurons were treated with hemin (10–60 μM) for 1 h **(C,D)** Primary cultured cortical neurons were treated with hemin (30 μM) plus afatinib (10 nM) for 1h. Western blot assay was employed to measure phosphorylated EGFR (p-EGFR) **(A,C)**, and phosphorylated AKT (p-AKT) **(B,D)**. Each lane contained 30 μg protein for all experiments. Graphs show statistical results from relative optical density of bands on the blots. Values are the mean ± S.E.M. (n = 3/each group). *p < 0.05 statistically significant in the hemin groups compared with the control groups; ^#^P < 0.05 in hemin plus afatinib compared with hemin alone by one-way ANOVA followed by the LSD test as post-hoc method.

The involvement of EGFR signaling in the hemin-induced neurotoxicity was investigated by measuring p-EGFR levels in primary cultured cortical neurons. We found that 1-h incubation of hemin (10–60 μM) concentration-dependently increased EGFR phosphorylation (Figure 2A). EGFR phosphorylation was evident when cells were treated with 30 μM hemin and maintained elevated with 60 μM hemin (Figure 2A). Similarly, incubation of hemin for 1 h significantly elevated p-AKT levels in a concentration-dependent manner (Figure 2B). Co-incubation of afatinib (10 nM) significantly attenuated hemin-induced EGFR phosphorylation (Figure 2C) and AKT phosphorylation (Figure 2D), indicating hemin indeed activated EGFR-AKT signaling in primary cultured cortical neurons.

**FIGURE 3:**
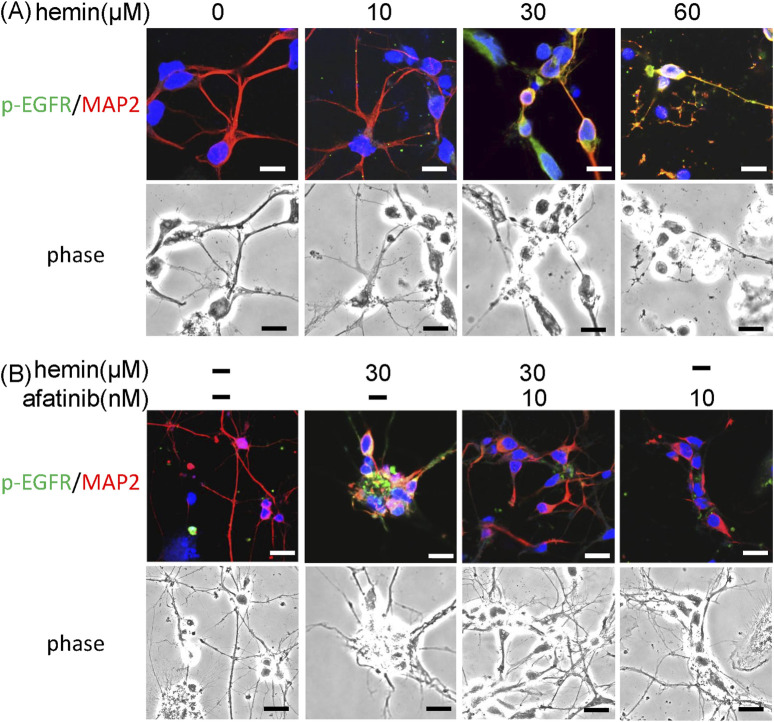
Effects of afatinib on hemin-induced impairment of neurite outgrowth. **(A)** Primary cultured cortical neurons were treated with hemin (10–60 μM) for 1 h. Representative immunofluorescent data show concentration-dependent damages of hemin on neurite outgrowth. **(B)** Primary cultured cortical neurons were treated with hemin (30 μM) plus afatinib (10 nM) for 1 h. The neurites were immunostained with phosphorylated EGFR (p-EGFR) and MAP-2. Calibration: 10 μm. The results were duplicated.

Furthermore, we investigated the effect of afatinib on hemin-induced morphological changes in primary cultured cortical neurons. Compared with the vehicle-treated cells, immunofluorescent staining data showed that hemin concentration-dependently (10–60 μM) increased p-EGFR immunoreactivity and damaged neurite outgrowth (Figure 3A). Incubation of hemin (10 μM) for 8 h did not cause significant changes in the neurite outgrowth. However, higher concentrations of hemin (30–60 μM) induced strong p-EGFR immunoreactivity. At the same time, hemin caused focal bead-like swellings, neuritic beading and discontinuities of neurites in primary cultured cortical neurons (Figure 3A). Co-incubation with afatinib (10 nM) attenuated hemin-induced elevation in p-EGFR immunoreactivity and impairment in neurite outgrowth (Figure 3B), suggesting that the EGFR signaling pathway is responsible for the hemin-induced damage to neurite outgrowth in primary cultured cortical neurons.

### Afatinib attenuated hemin-induced neuroinflammation, HO-1 expression and programmed cell death

**FIGURE 4:**
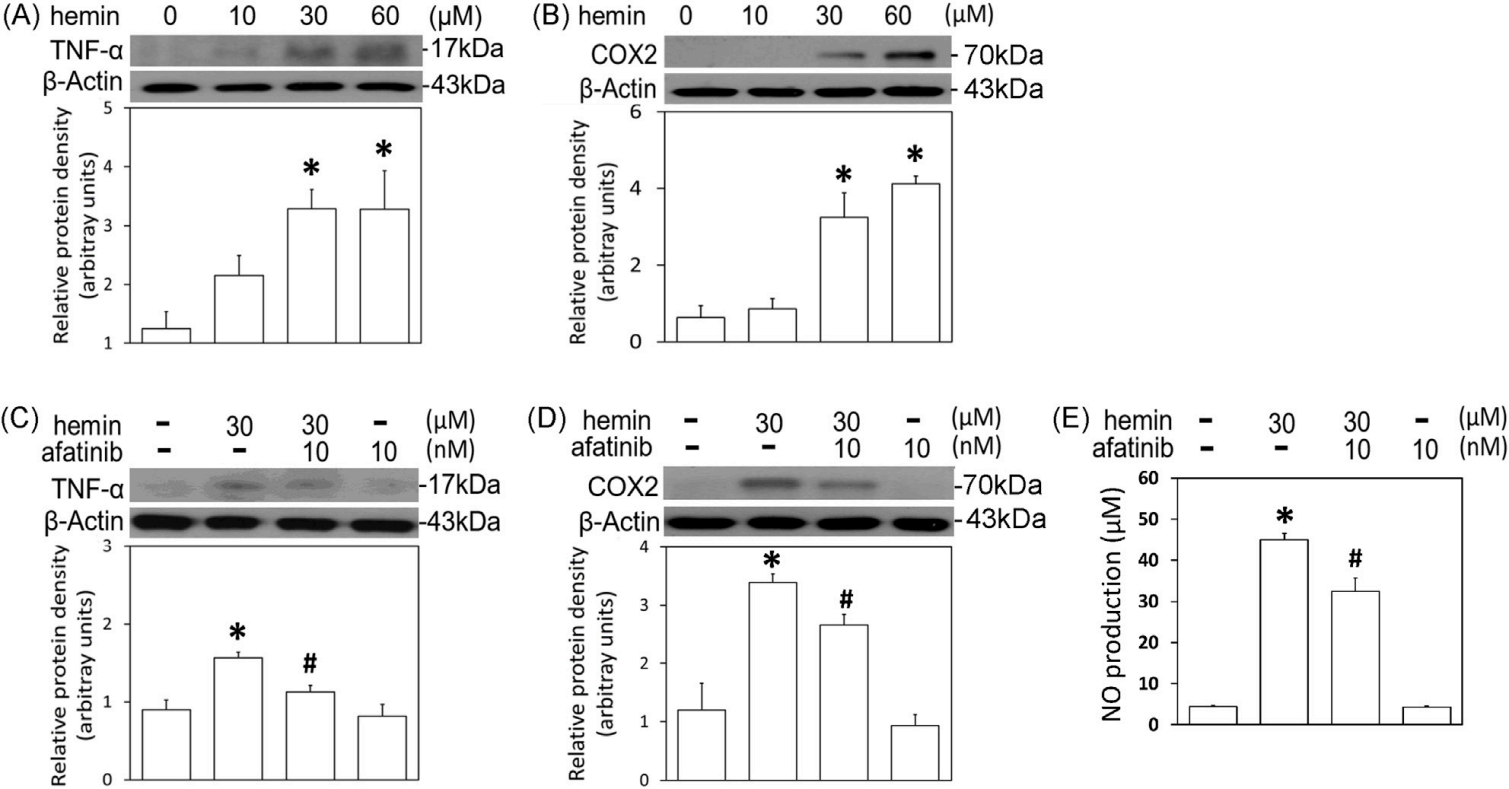
Effects of afatinib on hemin-induced neuroinflammation. **(A**, **B)** Primary cultured cortical neurons were treated with hemin (10–60 μM) for 16 h. **(C,D)** Primary cultured cortical neurons were treated with hemin (30 μM) plus afatinib (10 nM) for 16 h. Western blot assay was employed to measure TNF-α **(A,C)** and COX2 **(B,D)**. Each lane contained 30 μg protein for all experiments. Graphs show statistical results from relative optical density of bands on the blots. **(E)** Primary cultured cortical neurons were treated with hemin (30 μM) plus afatinib (10 nM) for 24 h. The levels of NO in culture medium were measured using Griess reaction. Values are the mean ± S.E.M. (n = 3/each group). *p < 0.05 statistically significant in the hemin groups compared with the control groups; ^#^P < 0.05 in hemin plus afatinib compared with hemin alone by one-way ANOVA followed by the LSD test as post-hoc method.

**FIGURE 5:**
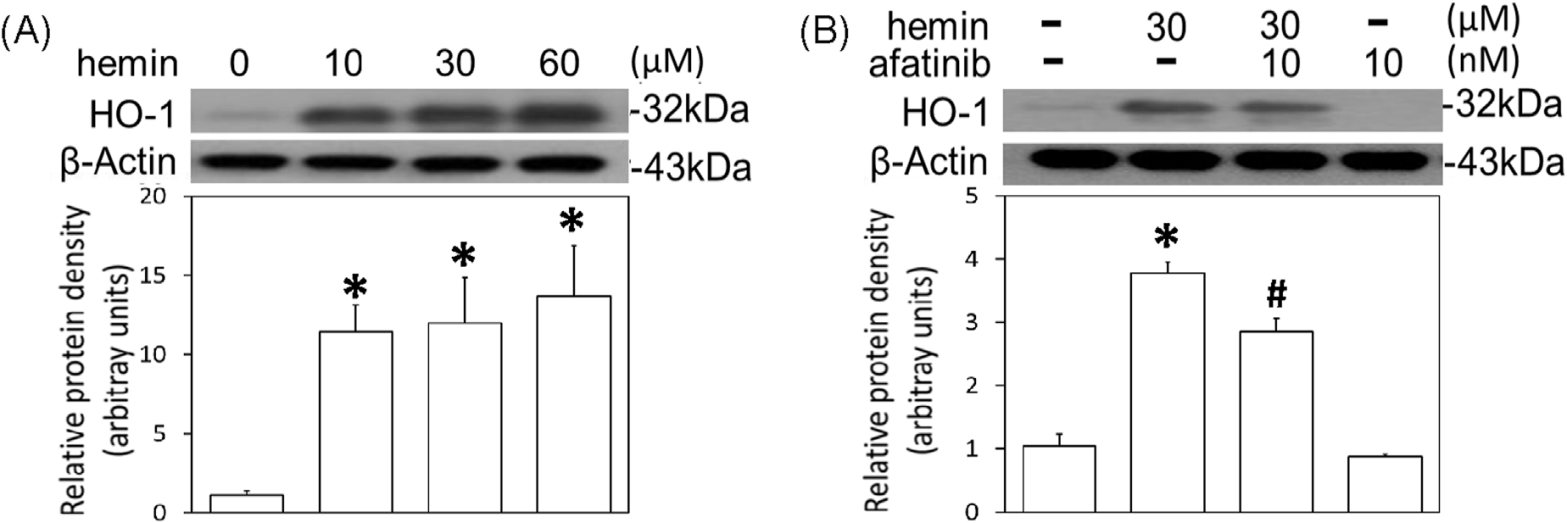
Effects of afatinib on hemin-induced HO-1 expression. **(A)** Primary cultured cortical neurons were treated with hemin (10–60 μM) for 16 h. **(B)** Primary cultured cortical neurons were treated with hemin (30 μM) plus afatinib (10 nM) for 16 h. Western blot assay was employed to measure HO-1. Each lane contained 30 μg protein for all experiments. Graphs show statistical results from relative optical density of bands on the blots. Values are the mean ± S.E.M. (n = 3/each group). *p < 0.05 statistically significant in the hemin groups compared with the control groups; ^#^P < 0.05 in hemin plus afatinib compared with hemin alone by one-way ANOVA followed by the LSD test as post-hoc method.

To further confirm the involvement of EGFR-AKT signaling in ICH, afatinib was employed to block hemin-induced activation of EGFR-TK signaling and related neurotoxicity. First, we established hemin-induced neuroinflammation in primary cultured cortical neurons. Western blot assay showed that a 16-hr incubation of hemin concentration-dependently (10–60 μM) increased TNF-α (Figure 4A) and COX2 protein levels (Figure 4B) in primary cultured cortical neurons. Co-incubation with afatinib (10 nM) prevented hemin (30 μM)-induced elevations in TNF-α (Figure 4C) and COX2 (Figure 4D) as well as NO production in the culture medium of treated neurons (Figure 4E), suggesting that afatinib is capable of reducing hemin-induced neuroinflammation. At the same time, we investigated the effect of afatinib on HO-1 expression (an enzyme catalyzing hemin). Western blot assay showed that a 16-h incubation of hemin (10–60 μM) increased HO-1 levels (Figure 5A). Co-incubation with afatinib (10 nM) prevented hemin (30 μM)-induced elevation in HO-1 (Figure 5B), suggesting that afatinib is capable of reducing hemin-induced HO-1 expression.

**FIGURE 6:**
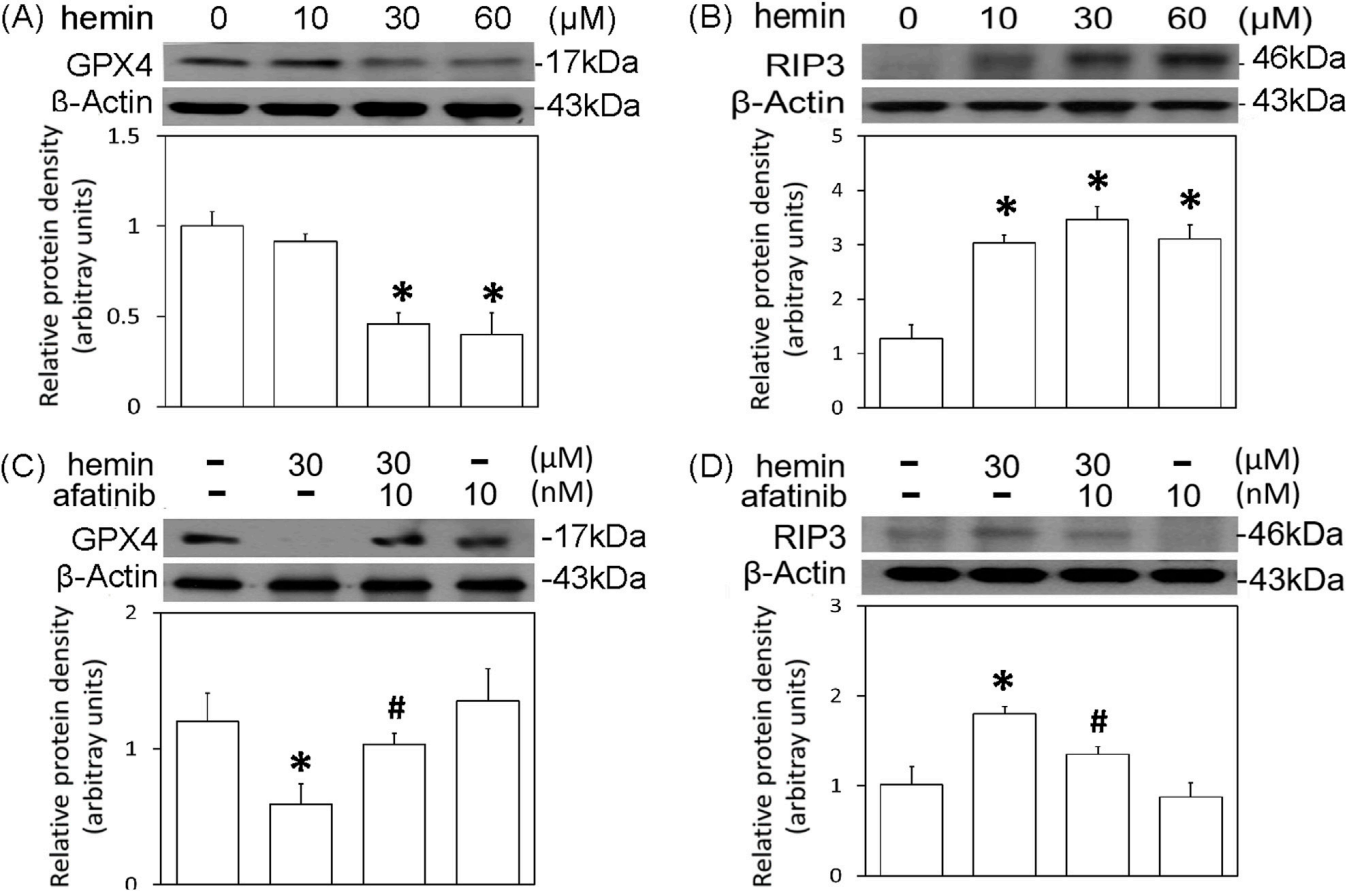
Effects of afatinib on hemin-induced programmed cell death. **(A,B)** Primary cultured cortical neurons were treated with hemin (10–60 μM) for 16 h. **(C,D)** Primary cultured cortical neurons were treated with hemin (30 μM) plus afatinib (10 nM) for 16 h. Western blot assay was employed to measure GPX4 **(A,C)** and RIP3 **(B,D)**. Each lane contained 30 μg protein for all experiments. Graphs show statistical results from relative optical density of bands on the blots. Values are the mean ± S.E.M. (n = 3/each group). *p < 0.05 statistically significant in the hemin groups compared with the control groups; ^#^P < 0.05 in hemin plus afatinib compared with hemin alone by one-way ANOVA followed by the LSD test as post-hoc method.

The cell death mechanisms underlying hemin-induced neurotoxicity were investigated by measuring GPX4 (a biomarker of ferroptosis) and receptor-interacting protein 3 (RIP3, a biomarker of necroptosis). Western blot assay demonstrated that hemin concentration-dependently (10–60 μM) reduced GPX4 (Figure 6A) and increased RIP3 (Figure 6B). Co-incubation with afatinib (10 nM) inhibited the hemin (30 μM)-induced reduction in GPX4 (Figure 6C) and elevation in RIP3 (Figure 6D). These data indicate that afatinib is capable of reducing hemin-induced ferroptosis and necroptosis.

## Discussion

In the present study, the EGFR-TK signaling was involved in the pathophysiology of ICH in several areas. First, hemin-induced neurotoxicity was demonstrated by neuronal death, neurite outgrowth impairment, neuroinflammation, HO-1 expression, ferroptosis and necroptosis. Furthermore, hemin consistently activated EGFR-AKT signaling in primary cultured cortical neurons. Finally, afatinib significantly attenuated hemin-induced EGFR-AKT activation and neurotoxicity. Taken together, these data suggest that the EGFR-AKT signaling may be a druggable target when developing therapies for ICH.

A pathological role of EGFR signaling has been demonstrated in CNS neurodegenerative diseases, including SCI, AD, brain ischemia and subarachnoid hemorrhage [10, 13–15, 19] but not ICH. In the present study, we are the first to show the involvement of EGFR in the pathophysiology of ICH. To mimic ICH-related neurotoxicity, several *in vitro* studies used PC12 cells and primary cortical neurons subjected to hemin ranging from 30 μM to 50 mM [21, 23–25]. Furthermore, an animal study reported high micromolar (≈390 μM) hemin in the hematomas [26]. In the present study, hemin (10–60 μM) was found to induce concentration-dependent increases in EGFR-AKT phosphorylation and neurotoxicity in primary cultured cortical neurons. Consistent with Zhou’s study [25], we chose 30 μM hemin to further delineate the involvement of EGFR-AKT signaling in ICH by studying the effect of afatinib, a second-generation EGFR-TKI for lung cancers [7], in primary cultured cortical neurons. Due to its covalent bonding to the EGFR-AKT activation site [27], nanomolar range (1 and 10 nM) of afatinib was found to effectively attenuate EGFR activation in cancer cells [7, 27]. Similar to Chen’s studies [15], we used 10 nM afatinib in the present study to successfully block hemin-induced EGFR-AKT signaling and neurotoxicity in primary cultured cortical neurons.

A “vicious cycle” containing oxidative stress, protein aggregation and cell death has been proposed for the pathophysiology of CNS neurodegenerative diseases; neuroinflammation is at the center of this “vicious cycle” [28]. Both clinical [29] and non-clinical studies [1, 2, 30] showed significant neuroinflammation in the ICH-affected brain tissues, including increases in inflammatory enzymes and proinflammatory cytokines. Our study supports this notion by demonstrating hemin-induced elevations in TNF-α and COX2 expression, as well as NO production. Similar to the afatinib-induced anti-inflammation in the OGD model [15], the present study using an ICH model demonstrated that afatinib attenuated hemin-induced neuroinflammation, suggesting that afatinib is anti-inflammatory in ICH.

Hemin is reportedly an HO-1 inducer [31, 32] to catalyze the degradation of heme/hemin [3, 33] and thus release iron [34] which is known as a Fenton’s reagent [35]. In the present study, hemin consistently elevated HO-1 expression which elevated iron levels that overproduced free radicals and oxidative injury [1]. Accordingly, in addition to anti-oxidant therapies [24, 36, 37], HO-1 inhibitors appear to be beneficial to ICH. Our study supports this notion that afatinib may exert its neuroprotective effect via attenuating HO-1 expression in ICH.

A significant body of literature has demonstrated several cell death mechanisms in ICH, including ferroptosis [21, 38] which is a programmed cell death related to iron metabolism [39–41]. Consistently, we identified hemin-induced ferroptosis in primary cultured cortical neurons. Moreover, we demonstrated that afatinib is capable of blocking hemin-induced ferroptosis. This may be due to afatinib-induced inhibition of hemin-elevated HO-1 expression which reduced iron accumulation, prevented Fenton’s reaction and then attenuated hemin-induced ferroptosis. In addition, we detected hemin-induced necroptosis, which was inhibited by afatinib, too. The afatinib-induced inhibition of necroptosis may be due to afatinib’s inhibition of hemin-elevated TNF-α levels because TNF-α reportedly initiates necroptosis and leads to RIP3 activation [42]. These data suggest that afatinib is capable of ameliorating ferroptosis and necroptosis in ICH.

In conclusion, the present study demonstrates that afatinib inhibited hemin-induced EGFR-AKT activation and neurotoxicity in primary cultured cortical neurons, suggesting that EGFR-AKT signaling is involved in the pathophysiology of ICH. Along with our previous study showing that afatinib inhibited OGD-induced neuroinflammation in astrocytes [15], it appears that EGFR-TKIs may be a novel repurposing drug for CNS neurodegenerative diseases, including ICH.

## Data Availability

The original contributions presented in the study are included in the article/supplementary material, further inquiries can be directed to the corresponding authors.

## References

[1] Shao Z Tu S Shao A. Pathophysiological mechanisms and potential therapeutic targets in intracerebral hemorrhage. Front Pharmacol (2019) 10:1079. 10.3389/fphar.2019.01079 31607923 PMC6761372

[2] Tschoe C Bushnell CD Duncan PW Alexander-Miller MA Wolfe SQ. Neuroinflammation after intracerebral hemorrhage and potential therapeutic targets. J Stroke (2020) 22:29–46. 10.5853/jos.2019.02236 32027790 PMC7005353

[3] Robinson SR Dang TN Dringen R Bishop GM. Hemin toxicity: a preventable source of brain damage following hemorrhagic stroke. Redox Rep (2009) 14:228–35. 10.1179/135100009x12525712409931 20003707

[4] Bai Q Liu J Wang G. Ferroptosis, a regulated neuronal cell death type after intracerebral hemorrhage. Front Cell Neurosci (2020) 14:591874. 10.3389/fncel.2020.591874 33304242 PMC7701249

[5] Magid-Bernstein J Girard R Polster S Srinath A Romanos S Awad IA Cerebral hemorrhage: pathophysiology, treatment, and future directions. Circ Res (2022) 130:1204–29. 10.1161/circresaha.121.319949 35420918 PMC10032582

[6] Wee P Wang Z. Epidermal growth factor receptor cell proliferation signaling pathways. Cancers (Basel) (2017) 9:52. 10.3390/cancers9050052 28513565 PMC5447962

[7] Liao BC Lin CC Yang JC. Second and third-generation epidermal growth factor receptor tyrosine kinase inhibitors in advanced nonsmall cell lung cancer. Curr Opin Oncol (2015) 27:94–101. 10.1097/cco.0000000000000164 25611025

[8] Varela L Garcia-Rendueles MER. Oncogenic pathways in neurodegenerative diseases. Int J Mol Sci (2022) 23:3223–6. 10.3390/ijms23063223 35328644 PMC8952192

[9] Romano R Bucci C. Role of EGFR in the nervous system. Cells (2020) 9:1887. 10.3390/cells9081887 32806510 PMC7464966

[10] Tavassoly O Del Cid Pellitero E Larroquette F Cai E Thomas RA Soubannier V Pharmacological inhibition of brain EGFR activation by a BBB-penetrating inhibitor, AZD3759, attenuates α-synuclein pathology in a mouse model of α-synuclein propagation. Neurotherapeutics (2021) 18:979–97. 10.1007/s13311-021-01017-6 33713002 PMC8423974

[11] Suh Y Obernier K Hölzl-Wenig G Mandl C Herrmann A Wörner K Interaction between DLX2 and EGFR regulates proliferation and neurogenesis of SVZ precursors. Mol Cell Neurosci (2009) 42:308–14. 10.1016/j.mcn.2009.08.003 19683576

[12] Birecree E Whetsell WO Jr Stoscheck C King LE Jr Nanney LB. Immunoreactive epidermal growth factor receptors in neuritic plaques from patients with Alzheimer's disease. J Neuropathol Exp Neurol (1988) 47:549–60. 10.1097/00005072-198809000-00006 3049945

[13] Mansour HM Fawzy HM El-Khatib AS Khattab MM. Potential repositioning of anti-cancer EGFR inhibitors in alzheimer's disease: current perspectives and challenging prospects. Neuroscience (2021) 469:191–6. 10.1016/j.neuroscience.2021.06.013 34139302

[14] Qu WS Tian DS Guo ZB Fang J Zhang Q Yu Z Inhibition of EGFR/MAPK signaling reduces microglial inflammatory response and the associated secondary damage in rats after spinal cord injury. J Neuroinflammation (2012) 9:642. 10.1186/1742-2094-9-178 PMC3418570 22824323

[15] Chen YJ Hsu CC Shiao YJ Wang HT Lo YL Lin AMY. Anti-inflammatory effect of afatinib (an EGFR-TKI) on OGD-induced neuroinflammation. Sci Rep (2019) 9:2516. 10.1038/s41598-019-38676-7 30792526 PMC6385176

[16] Planas AM Justicia C Soriano MA Ferrer I. Epidermal growth factor receptor in proliferating reactive glia following transient focal ischemia in the rat brain. Glia (1998) 23:120–9. 10.1002/(sici)1098-1136(199806)23:2<120::aid-glia3>3.0.co;2-a 9600380

[17] Peng DH Liu YY Chen W Hu HN Luo Y. Epidermal growth factor alleviates cerebral ischemia-induced brain injury by regulating expression of neutrophil gelatinase-associated lipocalin. Biochem Biophysical Res Commun (2020) 524:963–9. 10.1016/j.bbrc.2020.02.025 32059851

[18] Yu Y Zhang X Han Z Zhao W Zhang L. Expression and regulation of miR-449a and AREG in cerebral ischemic injury. Metab Brain Dis (2019) 34:821–32. 10.1007/s11011-019-0393-9 30773606

[19] Nakano F Kanamaru H Kawakita F Liu L Nakatsuka Y Nishikawa H Epidermal growth factor receptor mediates neuronal apoptosis after subarachnoid hemorrhage in mice. Stroke (2023) 54:1616–26. 10.1161/strokeaha.122.041977 37154060

[20] Min H Choi B Jang YH Cho IH Lee SJ. Heme molecule functions as an endogenous agonist of astrocyte TLR2 to contribute to secondary brain damage after intracerebral hemorrhage. Mol Brain (2017) 10:27. 10.1186/s13041-017-0305-z 28646881 PMC5483261

[21] Zille M Karuppagounder SS Chen Y Gough PJ Bertin J Finger J Neuronal death after hemorrhagic stroke *in vitro* and *in vivo* shares features of ferroptosis and necroptosis. Stroke (2017) 48:1033–43. 10.1161/strokeaha.116.015609 28250197 PMC5613764

[22] Zhou J Du T Li B Rong Y Verkhratsky A Peng L. Crosstalk between MAPK/ERK and PI3K/AKT signal pathways during brain ischemia/reperfusion. ASN Neuro (2015) 7:1759091415602463. 10.1177/1759091415602463 26442853 PMC4601130

[23] Karuppagounder SS Alim I Khim SJ Bourassa MW Sleiman SF John R Therapeutic targeting of oxygen-sensing prolyl hydroxylases abrogates ATF4-dependent neuronal death and improves outcomes after brain hemorrhage in several rodent models. Sci Transl Med (2016) 8:328ra29. 10.1126/scitranslmed.aac6008 PMC5341138 26936506

[24] Duan L Zhang Y Yang Y Su S Zhou L Lo PC Baicalin inhibits ferroptosis in intracerebral hemorrhage. Front Pharmacol (2021) 12:629379. 10.3389/fphar.2021.629379 33815110 PMC8017143

[25] Zhou YF Zhang C Yang G Qian ZM Zhang MW Ma J Hepcidin protects neuron from hemin-mediated injury by reducing iron. Front Physiol (2017) 8:332. 10.3389/fphys.2017.00332 28588503 PMC5440571

[26] Letarte PB Lieberman K Nagatani K Haworth RA Odell GB Duff TA. Hemin: levels in experimental subarachnoid hematoma and effects on dissociated vascular smooth-muscle cells. J Neurosurg (1993) 79:252–5. 10.3171/jns.1993.79.2.0252 8331409

[27] Solca F Dahl G Zoephel A Bader G Sanderson M Klein C Target binding properties and cellular activity of afatinib (BIBW 2992), an irreversible ErbB family blocker. The J Pharmacol Exp Ther (2012) 343:342–50. 10.1124/jpet.112.197756 22888144

[28] Zhang W Xiao D Mao Q Xia H. Role of neuroinflammation in neurodegeneration development. Signal Transduction Targeted Ther (2023) 8:267. 10.1038/s41392-023-01486-5 PMC10336149 37433768

[29] Almarghalani DA Sha X Mrak RE Shah ZA. Spatiotemporal cofilin signaling, microglial activation, neuroinflammation, and cognitive impairment following hemorrhagic brain injury. Cells (2023) 12:1153. 10.3390/cells12081153 37190062 PMC10137307

[30] Mayne M Ni W Yan HJ Xue M Johnston JB Del Bigio MR Antisense oligodeoxynucleotide inhibition of tumor necrosis factor-alpha expression is neuroprotective after intracerebral hemorrhage. Stroke (2001) 32:240–8. 10.1161/01.str.32.1.240 11136943

[31] Ouyang Y Li D Wang H Wan Z Luo Q Zhong Y MiR-21-5p/dual-specificity phosphatase 8 signalling mediates the anti-inflammatory effect of haem oxygenase-1 in aged intracerebral haemorrhage rats. Aging Cell (2019) 18:e13022. 10.1111/acel.13022 31400088 PMC6826124

[32] Le LL Li XY Meng D Liang Q Wang X Li N Heme oxygenase-1 mediated memorial and revivable protective effect of ischemic preconditioning on brain injury. CNS Neurosci Ther (2013) 19:963–8. 10.1111/cns.12152 23870531 PMC6493422

[33] Chen-Roetling J Lu X Regan RF. Targeting heme oxygenase after intracerebral hemorrhage. Ther Targets Neurol Dis (2015) 2:474. 10.14800/ttnd.474 25642455 PMC4310000

[34] Hu S Hua Y Keep RF Feng H Xi G. Deferoxamine therapy reduces brain hemin accumulation after intracerebral hemorrhage in piglets. Exp Neurol (2019) 318:244–50. 10.1016/j.expneurol.2019.05.003 31078524 PMC6588480

[35] Winterbourn CC. Toxicity of iron and hydrogen peroxide: the Fenton reaction. Toxicol Lett (1995) 82-83:969–74. 10.1016/0378-4274(95)03532-x 8597169

[36] Duan X Wen Z Shen H Shen M Chen G. Intracerebral hemorrhage, oxidative stress, and antioxidant therapy. Oxidative Med Cell Longevity (2016) 2016:1203285. 10.1155/2016/1203285 PMC4848452 27190572

[37] Zheng Y Li R Fan X. Targeting oxidative stress in intracerebral hemorrhage: prospects of the natural products approach. Antioxidants (Basel) (2022) 11:1811–24. 10.3390/antiox11091811 36139885 PMC9495708

[38] Zhang Y Khan S Liu Y Zhang R Li H Wu G Modes of brain cell death following intracerebral hemorrhage. Front Cell Neurosci (2022) 16:799753. 10.3389/fncel.2022.799753 35185473 PMC8851202

[39] Lu C Tan C Ouyang H Chen Z Yan Z Zhang M. Ferroptosis in intracerebral hemorrhage: a panoramic perspective of the metabolism, mechanism and theranostics. Aging Dis (2022) 13:1348–64. 10.14336/ad.2022.01302 36186133 PMC9466971

[40] Tang D Chen X Kang R Kroemer G. Ferroptosis: molecular mechanisms and health implications. Cell Res (2021) 31:107–25. 10.1038/s41422-020-00441-1 33268902 PMC8026611

[41] Li Q Han X Lan X Gao Y Wan J Durham F Inhibition of neuronal ferroptosis protects hemorrhagic brain. JCI Insight (2017) 2:e90777. 10.1172/jci.insight.90777 28405617 PMC5374066

[42] Sun X Lee J Navas T Baldwin DT Stewart TA Dixit VM. RIP3, a novel apoptosis-inducing kinase. J Biol Chem (1999) 274:16871–5. 10.1074/jbc.274.24.16871 10358032

